# Identification of Novel SARS-CoV-2 Drug Targets by Host MicroRNAs and Transcription Factors Co-regulatory Interaction Network Analysis

**DOI:** 10.3389/fgene.2020.571274

**Published:** 2020-10-14

**Authors:** Rahila Sardar, Deepshikha Satish, Dinesh Gupta

**Affiliations:** ^1^Translational Bioinformatics Group, International Centre for Genetic Engineering and Biotechnology (ICGEB), New Delhi, India; ^2^Department of Biochemistry, Jamia Hamdard, New Delhi, India

**Keywords:** regulatory network, miRNA, SARS-CoV-2, hub genes, TFS

## Abstract

Understanding the host regulatory mechanisms opposing virus infection and virulence can provide actionable insights to identify novel therapeutics against severe acute respiratory syndrome coronavirus 2 (SARS-CoV-2). We have used a network biology approach to elucidate the crucial factors involved in host responses involving host–microRNA (miRNA) interactions with host and virus genes using recently published experimentally verified protein–protein interaction data. We were able to identify 311 host genes to be potentially targetable by 2,197 human miRNAs. These miRNAs are known to be involved in various biological processes, such as T-cell differentiation and activation, virus replication, and immune system. Among these, the anti-viral activity of 38 miRNAs to target 148 host genes is experimentally validated. Six anti-viral miRNAs, namely, hsa-miR-1-3p, hsa-miR-17-5p, hsa-miR-199a-3p, hsa-miR-429, hsa-miR-15a-5p, and hsa-miR-20a-5p, are previously reported to be anti-viral in respiratory diseases and were found to be downregulated. The interaction network of the 2,197 human miRNAs and interacting transcription factors (TFs) enabled the identification of 51 miRNAs to interact with 77 TFs inducing activation or repression and affecting gene expression of linked genes. Further, from the gene regulatory network analysis, the top five hub genes *HMOX1*, *DNMT1*, *PLAT*, *GDF1*, and *ITGB1* are found to be involved in interferon (IFN)-α2b induction, epigenetic modification, and modulation of anti-viral activity. The comparative miRNAs target identification analysis in other respiratory viruses revealed the presence of 98 unique host miRNAs targeting SARS-CoV-2 genome. Our findings identify prioritized key regulatory interactions that include miRNAs and TFs that provide opportunities for the identification of novel drug targets and development of anti-viral drugs.

## Introduction

Severe acute respiratory syndrome coronavirus 2 (SARS-CoV-2) is a single-stranded positive-sense RNA β-coronavirus of the Coronaviridae family and shares the highest similarity with SARS-CoV, which is the virus responsible for the 2003 SARS outbreak (Wang et al., 2020).

SARS-CoV-2 genome is ∼29–30 kb and is translated into 29 proteins, including structural and non-structural proteins. In SARS-CoV-2 genome, more than two-thirds of the genome constitutes orf1ab, encoding orf1ab polyproteins at 5’, whereas the other one-third is associated with genes encoding structural proteins that include surface (S), envelope (E), membrane (M), and nucleocapsid (N) proteins at the 3′ end genome. Moreover, the SARS-CoV-2 genome codes for six accessory proteins, encoded by ORF3a, ORF6, ORF7a, ORF7b, and ORF8 genes (Khailany et al., 2020).

Viruses are obligate intracellular parasites that use the host cellular machinery to replicate and propagate. RNA viruses tend to evolve rapidly by mutations, enabling its evasion from the host immune response. With evolution, the hosts also develop different ways to fight virus infection, which include innate immunity that provides the first line of defense against viral infections. The host cells have many receptors that recognized virus elements that led to the activation of the interferon system and cytokines (Nakhaei et al., 2009; Girardi et al., 2018). Coronaviruses are known to induce the activation of host pathways linked to stress, apoptosis, autophagy, and innate immunity (Fung and Liu, 2019).

MicroRNAs (miRNAs) are ∼19- to 24-nt non-coding RNAs that regulate gene expression by binding to target messenger RNAs (mRNAs). Several miRNAs play a key role in cell differentiation, development, pathogenesis, cellular growth, apoptosis, and disease progression (Lou et al., 2019). During viral infections, host miRNAs are involved in various signaling pathways modulating host–virus interactions. Host miRNAs regulate viral infectivity and transmission, activate anti-viral immune responses, and play a role in many viral diseases, such as HIV, herpesvirus, and Ebola, by downregulating the host genes (Bernier and Sagan, 2018).

Targeting the host factors has been proven to be useful for restricting viral infections and has a potential for the development of host-directed therapies (HDTs) (Kaufmann et al., 2018). It is predicted that host miRNAs target over two-thirds of all human genes (Friedman et al., 2009). The latest release of miRBase consists of 2,654 mature human miRNAs with the ability to target hundreds of different types of genes and proteins (Griffiths-Jones, 2006).

**FIGURE 1:**
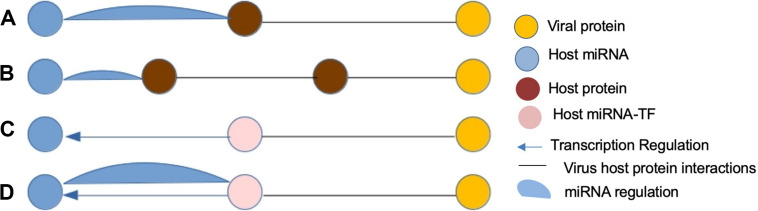
Four most anticipated patterns of host miRNA-mediated virus–host interactions (FFLs). **(A)** A viral protein and human miRNA have common human target. **(B)** A viral protein and human miRNA have a protein pair as common target. **(C)** A viral protein targeting a TF of a human miRNA. **(D)** A viral protein targeting a host protein forming a feedback loop to a human miRNA.

Transcription factors (TFs) are master regulators that regulate gene transcription by binding to the promoter region of the DNA-binding domains of target genes. Moreover, it is known that there is an interplay between host miRNAs and TFs (Tong et al., 2019) during viral infections as shown in Figure 1, and both are regulated in a coordinated fashion in various biological processes, such as cell proliferation, differentiation, and apoptosis, and many diseases through feed-forward loops (FFLs) and feedback loops (FBLs) (Tsang et al., 2007; Mohamed et al., 2019). Therefore, the identification of key regulatory miRNA–TF interactions can play a crucial role in the development of anti-viral therapies against SARS-CoV-2.

Recently, using computational tools and databases, few groups of scientists have identified potential host miRNAs that target SARS-CoV-2 genes involved in immune signaling pathways (Ahmed et al., 2020, p. 19; Khan et al., 2020). However, these are limited reports focused on the identification of host miRNA and lack information about their regulatory interactions especially with TFs. Therefore, in the present study, we have investigated miRNAs targeting host genes and its interactions with TFs exploiting the recently published SARS-CoV-2–protein interaction map (Gordon et al., 2020). Out of the 332 host genes interacting with the virus genes, 311 are found to be targeted by 2,197 human miRNAs and involved in various biological processes, such as T-cell differentiation, onco-miRNAs, toxicity, immune system, and viral replications. After an extensive literature survey, we were able to identify 38 miRNAs with anti-viral activity targeting 143 host genes. Six anti-viral miRNAs, namely, hsa-miR-1-3p, hsa-miR-17-5p, hsa-miR-199a-3p, hsa-miR-429, hsa-miR-15a-5p, and hsa-miR-20a-5p, were reported to play a role in respiratory diseases, such as influenza A, adenovirus 2, and respiratory syncytial virus (RSV), and were downregulated that can be used to design anti-viral drugs. From the comparative gene expression analysis, we identified 148 differentially regulated TFs in SARS, whereas in SARS-CoV-2 gene expression analysis, we identified that 48 and 52 TFs are expressed in A549 and normal human bronchial epithelial (NHBE) cell lines. Interestingly, among the differentially expressed TFs, only STAT1 and STAT2 are in the interactome connected with ORF3a and NSP7.

## Methodology

**FIGURE 2:**
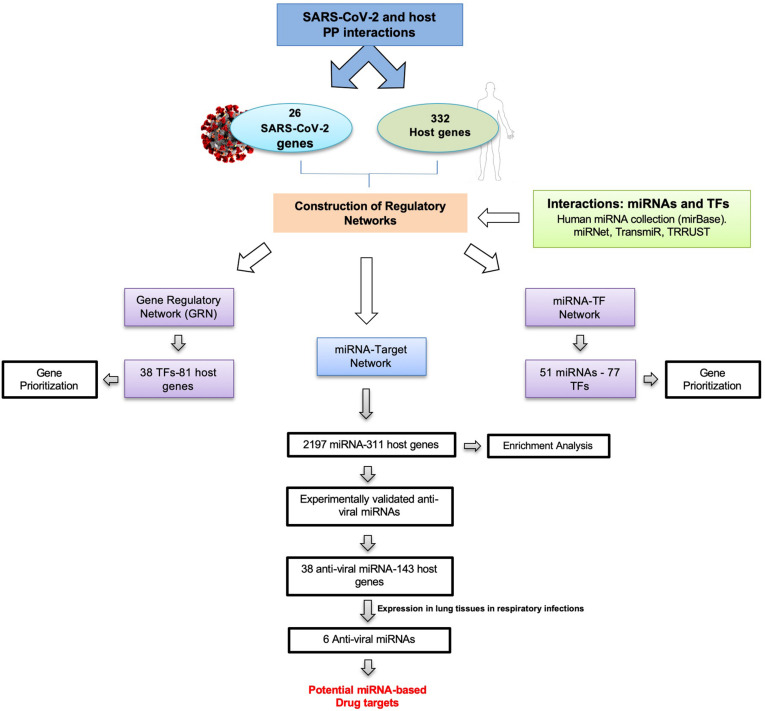
Pipeline design to identify novel drug targets in SARS-CoV-2 by generation and analysis of gene regulatory networks.

To identify miRNA–TF co-regulatory interactions in SARS-CoV-2, we have designed a pipeline as shown in Figure 2. Summarily, a list of host proteins interacting with virus proteins served as a starting dataset for the generation of different regulatory networks for the study.

### Virus–Human Interactome Dataset

We obtained the protein interaction map of SARS-CoV-2 and human proteins from a recent report of affinity-purification mass spectrometry experiments on the proteins (Gordon et al., 2020). The interactome consists of 26 SARS-CoV-2 (out of 29) proteins interacting with 332 human proteins (Supplementary Table S1). The host–virus interactome is a network where proteins are nodes and interactions between proteins are edges.

### Regulatory Interactome Analysis

For the identification of miRNA targeting host genes, its visualization and functional analysis were performed using the miRNet 1.0 server (Fan et al., 2016). The list of genes corresponding to the proteins identified to interact with the virus proteins was analyzed using miRNet to identify host miRNAs regulating the genes. A list of TFs interacting with the miRNAs was generated with the help of the miRNet and TransmiR v2.0 database (Tong et al., 2019). The hub genes were prioritized using network topological property, i.e., degree from the interaction network.

To identify miRNAs targeting SARS-CoV-2 genes, we performed miRNA target predictions using miRanda (version 3.3a) with an energy threshold of -20 kcal/mol (Betel et al., 2007). We downloaded a complete list of all the available mature human miRNAs from miRBase (Release 22.1) (Griffiths-Jones, 2006) and surveyed the literature to identify experimentally validated anti-viral miRNAs among the other miRNAs. To identify and compare miRNAs targeting other respiratory viruses (influenza A, measles, SARS-CoV related), we used miRTarP (Shu et al., 2018).

All human TFs were retrieved from the latest version of AnimalTFdb 3.0, which consists of 1,665 TFs classified into 73 TF families. Regulatory interactions (TF-target) from experimental evidences were extracted from TRRUST (version 2) database that contains 8,444 TF-target regulatory relationships of 800 human TFs (Han et al., 2018).

### Gene Expression Analysis

To identify miRNAs expressed in the lung tissues, we used the Tissue Atlas database (Ludwig et al., 2016). For the gene expression analysis of host genes, we obtained two datasets, namely, a microarray dataset for SARS-CoV and an RNA-seq dataset for SARS-CoV-2, from the NCBI GEO database and ViPR host factor data search (Pickett et al., 2012). The first dataset SARS-CoV (GSE17400) (Yoshikawa et al., 2010) is derived from mock-infected Calu-3 subclone 2B4 cells, and SARS-CoV-2 (GSE147507) gene expression datasets are based on the samples derived from the infected primary human lung epithelium (NHBE) and transformed lung alveolar (A549) cells (Blanco-Melo et al., 2020a, b, p. 19). The microarray data of SARS-CoV were normalized by Robust Multi-chip Average (RMA) and SARS-CoV-2 RNA-seq data by DESeq2 R package.

## Results

### Regulatory Interactome of Host Genes

**FIGURE 3:**
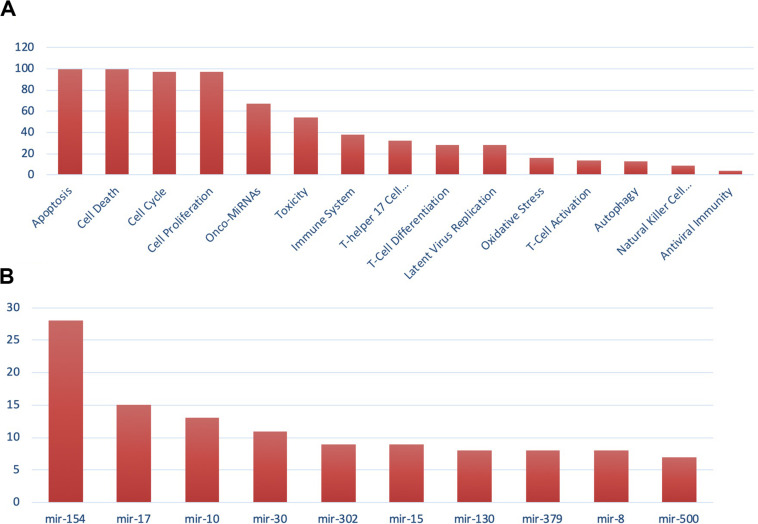
Gene set enrichment analysis showing **(A)** functional analysis of miRNAs and **(B)** miRNA family distribution.

**FIGURE 4:**
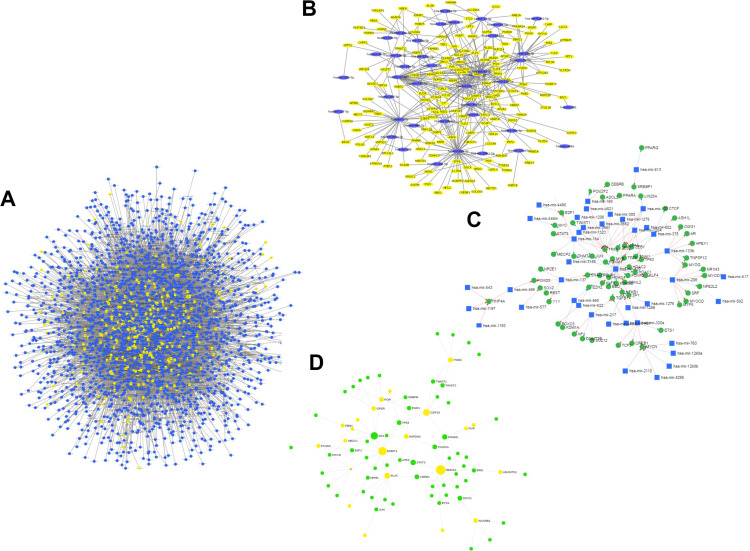
**(A)** miRNA-target (host genes) interactome. **(B)** Subnetwork of reported anti-viral miRNA (*n* = 38) interaction network. **(C)** miRNA–TF interactome for host genes (green nodes represent TFs, blue nodes represent miRNAs, and yellow nodes represent target host genes). **(D)** GRN of TF targeting host genes.

For the miRNA–host gene interactome analysis, we found that out of 332 host genes, 311 are found to be targeted by 2,197 human miRNAs (Supplementary Table S2). These genes are found to be involved in various biological processes, such as T-cell differentiation, onco-miRNAs, toxicity, regulation of Akt pathway, immune system, and others, as shown in Figure 3A. These miRNAs are distributed across 100 miRNA families, and the top 10 families among them are shown in Figure 3B. Further, from the degree prioritization, we identified the top five hub genes from the miRNA-target network as shown in Figure 4A of the miRNA-target network, and these are *DCAF7*, *G3BP1*, *SBNO1*, *PRRC2B*, and *GGCX*, which are reported to interact with SARS-CoV-2 NSP9, N, NSP12, and M proteins.

Further expression analysis of the miRNA resulted in 49 miRNAs expressed in lung tissues. From the extensive literature survey, we identified 38 out of 2,197 miRNAs targeting 143 number of host genes with anti-viral activity depicted in Figure 4B and (Supplementary Table S3). Interestingly, six, namely, hsa-miR-1-3p, hsa-miR-17-5p, hsa-miR-199a-3p, hsa-miR-429, hsa-miR-15a-5p, and hsa-miR-20a-5p, were reported to play a role in respiratory diseases, such as adenovirus 2, influenza A, and RSV, and were downregulated in lung tissues during viral infection but overexpressed in normal lung tissues (Zhu et al., 2014; Leon-Icaza et al., 2019).

To understand miRNA–TF co-regulation of host genes that interact with SARS-CoV-2 proteins, we generated a miRNA–TF network that led to the identification of 51 miRNAs interacting with 77 TFs by either activation or repression as shown in Figure 4C. The hsa-mir-429 (reported to have anti-viral activity) and TP53 (a TF) were found to have a maximum degree of 28 and 12 in the miRNA–TF network, respectively. From the functional enrichment analysis, we identified the involvement of genes in various important biological pathways, including MAPK signaling, B-cell, Toll-like receptor signaling, and viral diseases, such as hepatitis C or measles.

### Gene Regulatory Network Analysis

Out of 332 host genes, 81 genes are involved in the gene regulatory network (GRN) interacting with 38 TFs (Supplementary Table S4). After degree prioritization, we were able to identify the top five hub genes, namely, *HMOX1*, *DNMT1*, *PLAT*, *GDF1*, and *ITGB1*, directly targeting ORF3a and ORF8 among SARS-CoV-2 proteins as shown in Table 1. These are reported in modulating several virus activities, such as anti-viral immunity, epigenetic modification, and others (Danesh et al., 2011; Espinoza et al., 2017; Schäfer and Baric, 2017), as shown in Figure 4D.

**TABLE 1:** Hub genes identified from gene regulatory network analysis.

Host gene	SARS-CoV-2 protein	Degree	Viral activity
*HMOX1*	ORF3a	18	Modulation of anti-viral immunity by heme oxygenase-1
*DNMT1*	ORF8	11	Epigenetic modification in SARS-CoV
*PLAT*	ORF8	8	Acute lung injury following an acute severe respiratory infection
*GDF15*	ORF8	8	Promotes HRV infection and virus-induced lung inflammation
*ITGB1*	ORF8	6	Induced by IFN-α2b in SARS-CoV

### Gene Expression Analysis

From the gene expression analysis of TF, we identified 148 TFs differentially regulated in SARS, whereas in the SARS-CoV-2 gene expression dataset, 48 and 52 TFs are expressed in A549 and NHBE cell lines. From the differentially regulated TFs in SARS-CoV-2 expression data, we found only two differentially expressed TFs, namely, STAT1 and STAT2, to target *HMOX1* and *SCRAB1* that interact with the ORF3a and NPS7 virus proteins (Supplementary Table S5).

### Comparative Regulatory Interaction Analysis in Other Respiratory Diseases

We identified 1,018 miRNAs to target SARS-CoV-2 genes, among which 98 miRNAs are predicted to target SARS-CoV-2 genes uniquely when compared with other viruses studied here (Supplementary Table S6). 547 miRNAs targeting SARS-CoV-2 genes are found to be common to those in SARS-CoV, influenza A, and measles. Interestingly, we observed that 717 miRNAs targeting SARS-CoV-2 genes are common with those targeting SARS-CoV genes, possibly due to high genome similarity (Xu et al., 2020; Zhang and Holmes, 2020, p. 2) (Supplementary Table S7). The highest common host miRNAs targeting SARS-CoV-2 genes as well as other viruses in the study are those targeting influenza A virus (874 miRNAs), as shown in Table 2. miRNA interacting TFs are preferentially targeted by viral proteins; therefore, we further explored and identified miRNA–TF co-regulatory interactions. From the analysis, we identified 34, 41, 66, and 45 TFs interacting with 21, 30, 43, and 33 in SARS-CoV-2, SARS-CoV, influenza, and measles, respectively (Supplementary Table S8).

**TABLE 2:** miRNA identification in SARS-CoV-2 and other related respiratory viruses.

Virus	Virus family	All miRNAs	Conserved miRNAs with SARS-CoV-2	Unique miRNA	Anti-viral activity	miRNA–TF interactions
SARS-CoV related^+^	Coronaviridae	1,515	717	53	32	30–41
Influenza A^+^	Orthomyxoviridae	2,075	874	227	38	43–66
SARS-CoV-2*	Coronaviridae	1,018	–	98	88	21–34
Measles^+^	Paramyxoviridae	1,573	696	42	23	33–45

*^+^Interactions retrieved from mirTarP. ^∗^Interactions retrieved using miRanda.*

## Discussion

Host-directed therapies is a recent approach targeting host cell factors that are required by a pathogen for replication (Kaufmann et al., 2018). Studies on SARS-CoV and Middle East respiratory syndrome (MERS) have already explored identifying potential host drug targets to block pathways and genes involved in coronavirus replication (Gassen et al., 2019). From the literature evidence, it is reported that TFs and miRNAs are two important master regulators controlling gene expression at the transcriptional and post-transcriptional levels. Studies also suggested that they play a role in multiple diseases by FFLs or FBLs (Zhang et al., 2015). Viruses tend to increase their genes expression by downregulating the host gene expression either co-transcriptionally in the nucleus or post-transcriptionally in the nucleus or cytoplasm. In herpesvirus, viral factors block the transcription initiation process by inhibiting TAF4. Targeting transcription process by SARS-CoV-2 could help in preventing the assembling of RNA polymerase II on host genes (Saçar Demirci and Adan, 2020). Genome-wide target predictions have previously shown that TFs are susceptible to regulation by miRNAs (Enright et al., 2003). Further, it is believed that miRNAs co-evolve with TFs, and the rapidly evolving TFs preferentially activate miRNAs (Chen and Rajewsky, 2007; Qiu et al., 2010). Thus, a direct relationship can be elucidated between the gene regulation networks controlled by TFs at the transcriptional level and those controlled by miRNAs at the post-transcriptional level. It has been suggested that miRNAs may provide genetic switch mechanisms to essentially inactivate the target genes by regulation of TF functioning and TF-mediated events (Chen et al., 2011). The active concert of these regulators in association to the regulatory FBLs orchestrates various cellular mechanisms. Understanding of miRNA–TF cross-talk during SARS-CoV-2 viral infection can be a more useful information to enhance our knowledge in this direction. The degree of expression of miRNAs is generally correlated in normal and diseased conditions. Many experiments have shown that during viral infection, the host–virus interactions involve overlapping target proteins or protein pairs among viral proteins and human miRNAs (Li et al., 2013). Therefore, the understanding of miRNA–TF interactions can be of relevance to SARS-CoV-2 research aimed at the development of novel therapeutics by modulation of specific TFs. In this study, the miRNA–TF network and the prioritized key interactions, generated on the basis of previous experiments and our predictions, provide useful information about such targetable interactions. From the miRNA–TF interactome, we identified 51 miRNAs interacting with 77 TFs involved in various biological pathways, such as MAPK signaling, B-cell, and Toll-like receptor signaling. It may be speculated that these interactions are also conserved in coronavirus disease 2019 (COVID-19) infection, used by SARS-CoV-2 to evade host immune responses.

During viral infections, anti-viral defense mechanism gets activated by the expression of interferons (IFNs) that are regulated by IFN-regulatory factors (IRF) family and STAT family TFs (Chiang and Liu, 2019). From the gene regulatory analysis, we identified STAT1 and STAT2 to be interacting with *HMOX1* and *SCRAB1* genes, which further interact with ORF3a and NPS7 in the SARS-CoV-2 PPI network. From the gene expression analysis, these TFs are found to be upregulated during SARS-CoV-2 infection at 24 h, confirming their role in anti-viral host defense by activation of many IFN stimulated genes (ISGs) as observed previously in SARS-CoV infection (Yoshikawa et al., 2010).

Mammalian cells inhibit virus infection by targeting transcripts with cellular miRNA (Song et al., 2010). The first evidence came from the observation that human miR-32 could limit the replication of primate foamy virus type 1 (PFV-1) in cells (Lecellier, 2005). Further, the liver-specific miR-122 was unexpectedly found to enhance the replication of the hepatitis C virus, whereas miR-199a-3p, miR-210, and miR-125a-5p were reported to suppress hepatitis B virus (HBV) replication (Jopling, 2005). In other virus systems, miR-101 was shown to suppress herpes simplex virus type 1 (HSV-1) propagation, whereas miR-3232, miR-491, and miR-654 were shown to inhibit the influenza virus (Lecellier, 2005; Potenza et al., 2011; Zheng et al., 2011).

In the present study, we generated and analyzed regulatory networks involving interacting miRNAs and TFs, targeting host genes based on information from a recently published host–virus interactome data. From the network analysis, we identified 2,197 miRNAs targeting host genes that interact with SARS-CoV-2 proteins. Among them, we identified 38 miRNAs targeting 143 host genes with reported anti-viral activity. Interestingly, six anti-viral miRNAs, namely, hsa-miR-1-3p, hsa-miR-17-5p, hsa-miR-199a-3p, hsa-miR-429, hsa-miR-15a-5p, and hsa-miR-20a-5p, are found to be downregulated post-infection in various viral respiratory diseases infecting the lungs, whereas in normal cases, these miRNAs are overexpressed. Clinical investigations have revealed that patients with cardiac diseases, hypertension, or diabetes who are administered with angiotensin-converting enzyme 2 (ACE2)-enhancing drugs including inhibitors and blockers exhibited elevated expression of ACE2; thus, the risk of getting the SARS-CoV-2 infection increased manifolds in such patients (Fang et al., 2020). Among the identified miRNAs, hsa-mir-9-5p was recently reported to target the 3’ UTR of ACE2 (Chen and Zhong, 2020), and hsa-mir-27b-3p plays a regulatory role in ACE2 signaling (Chen et al., 2015). Thus, a strong correlation between miRNA hsa-mir-27b-3p and ACE2 is established, but further experimental validation in SARS-CoV-2 infection may confirm this finding. Post-infection changes in the cellular miRNA expression profile are well reported (Triboulet et al., 2007; Cameron et al., 2008; Wang et al., 2008). These changes in cellular miRNA expression may be specifically induced by a given virus to create a more favorable intracellular environment for viral replication. On the contrary, these changes could also represent another aspect of the host cell innate immune response, triggered by a viral challenge and may inhibit virus replication (Wang et al., 2008).

To the best of our knowledge, our study is the first-ever attempt to identify key gene regulators and their co-regulation involving immune and MAPK signaling pathways, which may be explored for designing drug targets against SARS-CoV-2.

## Data Availability Statement

Publicly available datasets were analyzed in this study. This data can be found here: GEO: https://www.ncbi.nlm.nih.gov/geo/query/acc.cgi?acc=GSE17400 and https://www.ncbi.nlm.nih.gov/geo/query/acc.cgi?acc=GSE147507.

## Author Contributions

DG and RS conceptualized the study and prepared the manuscript. RS, DG, and DS carried out the computational studies and performed the analysis. All the authors reviewed and approved the final version.

## Conflict of Interest

The authors declare that the research was conducted in the absence of any commercial or financial relationships that could be construed as a potential conflict of interest.

## Supplementary Material

TABLE S1 Host–SARS-CoV-2 interaction data.

TABLE S2 List of miRNA targeting host genes identified using the miRnet server.

TABLE S3 List of reported anti-viral miRna interactions.

TABLE S4 GRN interactions.

TABLE S5 Gene expression data of SARS-CoV and SARS-CoV-2.

TABLE S6 miRNA identification in SARS-CoV-2 using miRanda.

TABLE S7 miRNA identification in SARS-CoV, influenza, and measles from miRtarP.

TABLE S8 miRNA–TF interaction analysis in respiratory viruses.
